# The Effect of Support Surfaces on the Incidence of Pressure Injuries in Critically Ill Patients: A Randomized Clinical Trial

**DOI:** 10.1155/2018/3712067

**Published:** 2018-12-18

**Authors:** Wesley Henrique Bueno de Camargo, Rita de Cassia Pereira, Marcos T. Tanita, Lidiane Heko, Isadora C Grion, Josiane Festti, Ana Luiza Mezzaroba, Cintia Magalhães Carvalho Grion

**Affiliations:** ^1^Post-graduate Student, Universidade Estadual de Londrina, Londrina, Brazil; ^2^Registered Nurse, Universidade Estadual de Londrina, Londrina, Brazil; ^3^Registered Nurse, Associação Evangélica Beneficente de Londrina, Londrina, Brazil; ^4^Medicine Graduate Student, Unicesumar, Maringá, Brazil; ^5^Professor, Department of Internal Medicine, Universidade Estadual de Londrina, Londrina, Brazil

## Abstract

**Purpose:**

To analyze whether a viscoelastic mattress support surface can reduce the incidence of stage 2 pressure injuries compared to a standard hospital mattress with pyramidal overlay in critically ill patients.

**Method:**

A randomized clinical trial with intention-to-treat analysis was carried out recruiting patients with Braden scale ≤14 on intensive care unit admission from April 2016 to April 2017. Patients were allocated into two groups: intervention group (viscoelastic mattress) and control group (standard mattress with pyramidal overlay). The level of significance adopted was 5%.

**Results:**

A total of 62 patients were included in the study. There was a predominance of males (53%) and the mean age was 67.9 (SD 18.8) years. There were no differences in clinical or severity characteristics between the patients in the control group and the intervention group. Pressure injuries occurred in 35 patients, with a median time of 7 days (ITQ 4–10) from admission. The frequency of pressure injuries was higher in the control group (80.6%) compared to the intervention group (32.2%; *p* < 0.001).

**Conclusions:**

Viscoelastic support surfaces reduced the incidence of pressure injuries in moderate or higher risk critically ill patients when compared to pyramidal support surfaces.

## 1. Introduction

Patient safety involves all studies, practices, and actions promoted by health institutions to reduce or eliminate the risks of unnecessary harm to health care [1]. In order to reduce adverse events related to health care, the World Health Organization (WHO) together with the Joint Commission International (JCI) has established global patient safety goals, which combine strategies focused on higher risk situations, among them the reduction in pressure injuries [2, 3].

The presence of pressure injuries has been considered an indicator of quality in health services, and efforts have been made to establish guidelines that orientate practices to reduce the problem. Pressure injuries are an important public health problem with great repercussions in different areas, present at all levels of care and that affect mainly, but not only, critically ill patients hospitalized in the intensive care unit (ICU) [4–6].

There is a possibility to obtain drastic reductions in the indices of pressure injuries with high investment in educational and preventive practices, improving patient quality of life [7]. The current scientific knowledge demonstrates that it is possible to avoid almost the entire development of pressure injuries.

Among the preventive measures, some studies suggest the adoption of special surfaces for pressure management (SSPMs) or support surfaces [8–13]. Support surfaces can be mattresses, overlays, or integrated bed systems. Various types of support surfaces are available with different mechanisms for pressure and shear relief [14]. Comparison of alternative foam mattresses for prevention of pressure injuries has showed conflicting results [15–19].

Viscoelastic foam is a type of porous polymer material that conforms in proportion to the applied weight. It is classified as a nonpowered reactive foam surface. Viscoelastic foam support surfaces can redistribute point pressure and consequently reduce pressure intensity to the body. These types of support surfaces may promote better body adaptation, a larger contact surface and more effective pressure reduction compared to other nonpowered surfaces [20]. The viscoelastic polyethylene-urethane mattress was associated with reduction of interface pressure by 20 to 30% compared with standard hospital mattress [21].

A recent network meta-analysis showed that there is moderate-certainty evidence that powered active air surfaces reduce pressure injury incidence compared to standard hospital mattresses. The same study showed low-certainty evidence that nonpowered support surface may reduce pressure injury incidence and that it is highly uncertain which support surface is better [22].

This study aims to analyze whether a viscoelastic mattress can reduce the incidence of stage 2 pressure injuries in critically ill patients hospitalized in an adult intensive care unit compared to a standard hospital mattress covered with a pyramidal overlay.

## 2. Methods

This study was done in accordance with the appropriate institutional review body and carried out with the ethical standards set forth in the Helsinki Declaration of 1975. The research was approved by the Committee for Ethics in Research Involving Humans of the Evangelical Beneficent Association of Londrina—AEBEL with the Certificate of Presentation for Ethical Appreciation (CAAE) no. 51644915.0.0000.5231 and Opinion no. 3/2015, of October 30, 2015. The study is registered at clinicaltrials.gov under identification no. NCT02844166.

A randomized clinical trial with intention-to-treat analysis was performed involving critically ill patients at moderate or higher risk for development of pressure injuries, that is, those presenting a Braden ≤14 scale at ICU admission in the period from April 2016 to April 2017.

According to the Braden scale, six subscales or risk factors are validated: (1) sensory perception, (2) moisture, (3) activity, (4) mobility, (5) nutrition, and (6) friction and shear. The total score varies from 6 to 23 points and patients are classified as: very high risk (scores less than or equal to nine), high risk (10 to 12 points), moderate risk (13 to 14 points), low risk (15 to 18 points), and without risk (19 to 23 points) [23].

The exclusion criteria were age less than 18 years, length of stay in the ICU for less than 24 hours, contraindication for the performance of the standard pressure injuries prevention measures of the institution, presence of pressure injuries at ICU admission, and absence of the informed consent term.

Randomization was performed using a computerized table, and patients were allocated into two groups. Patients in the intervention group were cared for according to the standard pressure injuries prevention measures of the institution and used a viscoelastic mattress as a bedding surface with the following characteristics: 5-centimeter layer of cold foam with a density of 40 and 7-centimeter layer of viscoelastic foam with a density of 60 (Sweet Pedic Hospitalar®) measuring 190 by 90 centimeters. Patients in the control group were cared for according to standard pressure injuries prevention measures of the institution and used a standard hospital mattress covered with a pyramidal overlay. The standard hospital mattress is a 12-centimeter cold foam with a density of 33 measuring 188 by 80 centimeters. The pyramidal overlay is a 5-centimeter layer of polyurethane foam density 33 whose surface looks like egg carton.

The institution's pressure injuries prevention measures for critically ill patients are to apply body moisturizer after bathing without massaging bony prominences or areas with hyperemia; inspect the skin in the cephalocaudal direction, especially the areas of bony prominences every 24 hours to observe hyperemia, dryness, heat, hardness, and maceration; rigorously evaluate whether the entire oral or enteral diet was ingested, taking into account the gastric residue and change in decubitus position every 2 hours; evaluate the need for application of dressings to protect bony protrusions from friction; and always use the traction to mobilize the patient in the bed.

As this was a nonpharmacological intervention, the blinding of the health team was not possible; however, the statisticians responsible for the analyses were blind regarding the identity of the treatments. After the randomization, identification data were collected—date of birth, gender, weight measured by the patient lift (Jack 250^®^), height measured by a tape measure, simplified acute physiology score 3 (SAPS 3), sequential organ failure assessment (SOFA) on ICU admission, diagnosis of ICU admission, and the Braden scale.

SAPS 3 is a prognostic score used to describe the severity of illness and to calculate predicted mortality. This score is made of 20 variables that measure the acute physiology in the first hour after ICU admission and previous health status [24]. SOFA score provides a continuous evaluation of organ dysfunction by considering variations in six variables that measure organ function during ICU stays. SOFA score can be calculated every 24 hours during ICU stay using the worst value for each period [25]. Both scores are translated for use in Brazil [26, 27].

Patients were followed until ICU discharge. During the patient follow-up, the following data were collected daily: physical examination for the detection and classification of pressure injuries, maximum vasopressor dose, and the Braden scale. The cumulative fluid balance was collected for the first 24 hours of ICU admission. Dates of discharge from the ICU and from the hospital were noted. The main outcome of the study was considered as the occurrence of a stage 2 pressure injuries. Secondary outcomes were time spent in the ICU and hospital.

The data were analyzed in the MedCalc program for Windows, version 9.3.2.0 (MedCalc Software, Mariakerke, Belgium). The level of significance adopted was 5%, and the confidence interval was 95%. In the statistical tests, the calculated values of *p* were presented, and values of *p* < 0.05 were considered significant.

To demonstrate a reduction of incidence of stage 2 pressure injuries in the intervention group, considering a significance level of 95%, power of 80%, ratio between exposed and unexposed of 1 : 1, frequency of outcome in the control group of 80%, and frequency of outcome in the intervention group of 30%, a sample size of 60 participants was calculated, 30 in each group.

In the descriptive statistics, the continuous quantitative variables were described after being assessed for normal distribution, using the Shapiro–Wilk test. For variables that presented normal distribution, means and standard deviations (SD) were calculated; for nonnormal distribution, medians and interquartile ranges (ITQ) (25th percentile and 75th percentile) were calculated. The nominal categorical variables were described as absolute and relative frequency (%). In the analytical statistics, categorical variables were compared using the Pearson's chi-square test (χ^2^) or Fisher's exact test in cases where more than 20% of the expected frequencies in the tables were less than five. For the comparison of two groups of continuous variables with independent samples, the Student's *t*-test was used for the variables with normal distribution. For cases where distribution was not normal, the Mann–Whitney test was applied. Kaplan–Meier curves were constructed and the log-rank test applied to compare the main outcome (pressure injuries stage 2) between the two study groups.

## 3. Results

**Figure 1:**
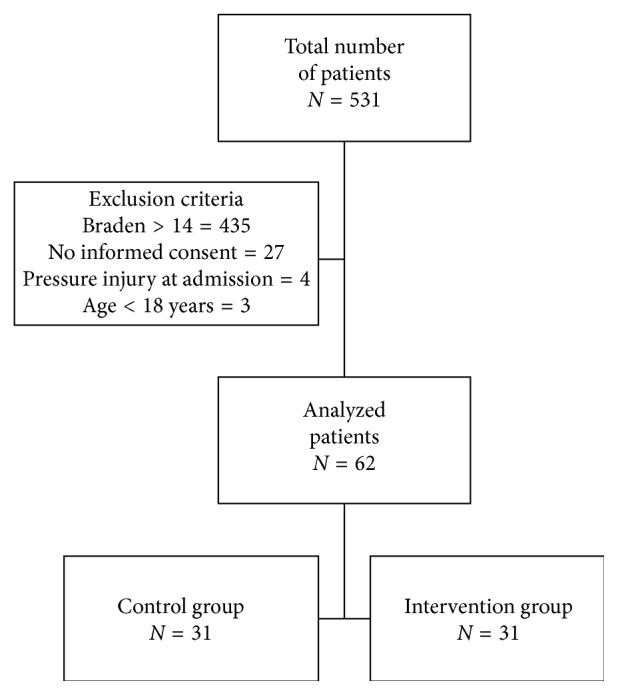
Flowchart of patients in the study. Control group = use of pyramidal mattress; intervention group = use of viscoelastic foam mattress.

A total of 531 patients were admitted to the ICU during the study period. Of these, 435 demonstrated a Braden scale >14 (low risk) at admission, four patients presented pressure injuries on ICU admission, 27 refused to participate, three patients were under 18 years old, and the remaining 62 patients met all the eligibility criteria and were analyzed in the study (Figure 1).

There was a predominance of the male gender (53.2%) and the mean age was 67.9 (SD 18.8) years. The mean SAPS 3 score was 69.4 (SD 14.9) and the mean SOFA score 8.3 (SD 4.2). The mean of the Braden scale was 10.8 (SD 1.7). The most frequent admission diagnoses were sepsis in 24 (38.71%) patients, followed by cardiac arrest in 6 (9.68%), congestive heart failure in 3 (4.84%), craniectomy due to intracranial hemorrhage in 3 (4.84%), and gastrointestinal bleeding in 3 (4.84%), among others. On admission to the ICU, 37 (59.7%) patients used a vasopressor, and the median accumulated fluid balance in the first 24 hours was 1,290 ml (ITQ 677–2.187).

The median length of stay in the ICU was 11.5 days (ITQ 7.5–22) and in the hospital 18.5 days (ITQ 10.5–29.0). There was no difference in the median length of stay in the ICU between the patients in the control group (15.0 days, ITQ: 8.5–23.5) and the intervention group (10 days, ITQ: 4.5–22.0; *p*=0.152).

The median length of hospital stay was higher among patients in the control group (22.0 days, ITQ: 14.5–37.5) compared to the intervention group (15 days, ITQ: 8.5–25.0; *p*=0.036). There were no differences in clinical characteristics or severity between the patients in the control group and the intervention group (Table 1).

**Figure 2:**
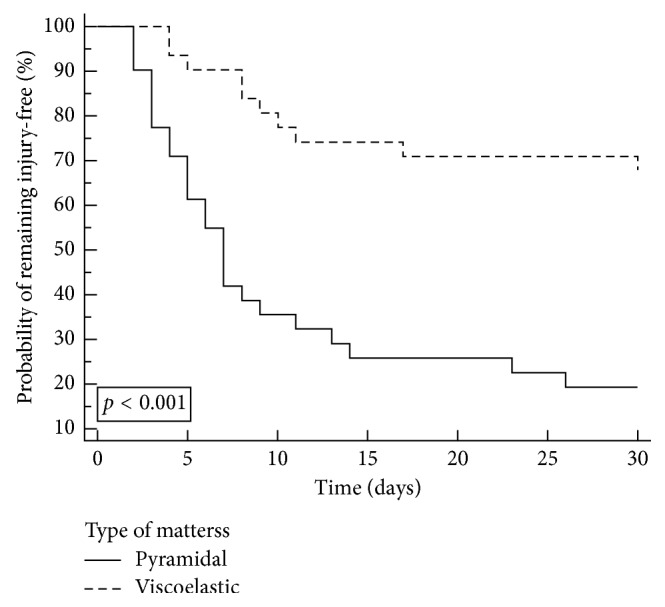
Kaplan–Meier curve for occurrence of pressure injuries in the study groups. *P*-value by log-rank test.

A pressure ulcer occurred in 35 patients, with a median time of 7 days (ITQ 4–10). A trend for a longer time for the occurrence of pressure injuries was observed in the intervention group (median 8.5 days; ITQ: 5.0–14.0) compared to the control group (median 6.0 days, ITQ: 3.0–8.0; *p*=0.088). The pressure injuries frequency was higher in the control group (80.6%) compared to the intervention group (32.2%; *p* < 0.001). There was a difference in the occurrence of pressure injuries between the study groups on the Kaplan–Meier curve (Figure 2).

## 4. Discussion

The present study demonstrated a protective effect of the viscoelastic foam surface compared to the pyramidal mattress in the occurrence of stage 2 pressure injuries in patients at moderate or higher risk for the development of these lesions. The incidence of wounds has a significant impact on patient quality of life, and many chronic wounds can be avoided, with appropriate diagnosis and treatment as well as alleviation of the suffering they cause [3, 28].

Several support surfaces are available to prevent pressure injuries. They can present different mechanisms for pressure relief including redistributing weight, mechanically alternating the pressure beneath body or a combination of these two mechanisms. Support surfaces are made from a variety of materials and identification of the optimum support surface remains a major challenge. A systematic review to compare effectiveness in pressure injury prevention with pressure-relieving support surfaces reports that patients at high risk of developing pressure ulcers should use higher-specification foam mattresses rather than standard hospital foam mattresses [15]. A recent network meta-analysis showed that powered active air mattress reduces pressure ulcer incidence but is associated with less comfort [22].

The integrity of skin plays an important role in pressure injury prevention. There are several intrinsic and extrinsic factors that can influence skin properties. In this context, the concept of microclimate was introduced and is of increasing interest in recent researches. Microclimate of local regions at risk for developing pressure injury consists of temperature, humidity, and airflow [29]. In the clinical setting, microclimate changes constantly due to medical procedures and devices. As an example, the less the occlusive materials are used, the greater evaporative capacity and the less the changes occur in the microclimate. Powered airflow support systems reduce humidity adjacent to the skin and induce less water accumulation when compared to standard mattress [30]. Viscoelastic and pyramidal foam do pressure redistribution and are nonpowered devices; both surfaces probably have similar effects on microclimate.

It is also noteworthy that although pressure injuries are related to adverse events linked to quality of care, the influence of intrinsic factors, that is, patient health conditions, may interfere in the appearance of the lesions, independent of the preventive measures and quality of care provided to these patients [31]. In this sense, it is possible to perceive that the act of hospitalization itself is one of the predisposing factors to the formation of pressure injuries, since the increase in functional loss and appearance of stress are evident. Immobility, age, nutritional status, emotional status, vascularization, systemic therapies, foreign bodies, skin characteristics, body weight, neurological factors, and different types of support surfaces adopted are factors that strongly influence the integrity of the skin [32].

The incidence of pressure injuries was high in the present study, and this finding can be attributed to multiple factors. The sample selected for the present study included patients at moderate or higher risk for the development of pressure injuries. The age factor is an element that potentiates the appearance of pressure injuries. During senescence, the effectiveness of the immune response as well as the skin turgor tends to decrease; due to a reduction in the production of collagen fibers and the percentage of water in the tissues, physical mobility may be altered, and there is greater bone fragility, among other factors. The mean body mass index between the study groups was compatible with a classification of overweight, and other authors describe the higher frequency of pressure injuries in overweight and obese patients [33].

The development of pressure injuries, especially those acquired in the hospital environment, is an important indicator of the quality of health care. Development of pressure injuries can have an important financial impact on the institutions, encouraging them to focus on prevention. Although the cost to prevent pressure injuries can importantly impact health-care services' budgets, the costs to treat a severe pressure ulcer can be substantially higher [34].

Decisions as to what support surface to use may be based on the assessment of how surfaces work and which is most appropriate for each patient. It is also important to consider cost-effectiveness when selecting support surfaces. For appropriate selection of the device based on the clinical conditions of the patient, professionals need to understand the properties, characteristics, and functionalities of the different support surfaces. With full evaluation and through risk scales, it is possible to classify patients according to the risk for pressure injury development. With these criteria, the professional can choose the most appropriate SSPM. Thus, a patient with a low risk of developing lesions could probably be safely accommodated on a pyramidal surface, while viscoelastic surfaces should be indicated for patients at higher risk.

In conclusion, from the results obtained in this clinical trial, viscoelastic support surfaces reduced the incidence of pressure injuries in moderate or higher risk critically ill patients when compared to pyramidal support surfaces.

## Figures and Tables

**Table 1:** Comparison of the clinical characteristics and prognostic scores of the groups of patients in the study.

	Total (*n*=62)	Group 1 (*n*=31)	Group 2 (*n*=31)	*p* value
Age (years), mean (SD)	67.9 (18.8)	71.5 (18.0)	64.2 (19.2)	0.127^*∗*^
Masculine, N (%)	33 (53.2)	15 (48.3)	18 (58.0)	0.305^†^
BMI, mean (SD)	27.6 (4.8)	27.1 (4.1)	28.0 (5.5)	0.496^*∗*^
Braden, mean (SD)	10.8 (1.7)	10.8 (1.7)	10.9 (1.7)	0.884^*∗*^
SAPS 3, mean (SD)	69.4 (14.9)	70.8 (15.2)	67.9 (14.7)	0.444^*∗*^
SOFA, mean (SD)	8.3 (4.2)	8.4 (4.5)	8.2 (3.9)	0.834^*∗*^
LOS ICU (days), median (ITQ)	11.5 (7.5–22.0)	15.0 (8.5–23.5)	10.0 (4.5–22.0)	0.152^‡^
LOS hospital (days), median (ITQ)	18.5 (10.5–29.0)	22.0 (14.5–37.5)	15.0 (8.5–25.0)	0.036^‡^

Group 1 = pyramidal; Group 2 = viscoelastic; ITQ: interquartile range; BMI: body mass index; SD: standard deviation; ICU: intensive care unit; SAPS 3: simplified acute physiology score; SOFA: sequential organ failure assessment; LOS: length of stay. ^*∗*^Student's *t*-test; ^†^chi-squared test; ^‡^Mann–Whitney test.

## Data Availability

The data used to support the findings of this study are available from the corresponding author upon request.
